# Influence of the Internal Structure Type of a Large-Area Lower Exhaust Workbench on Its Surface Air Distribution

**DOI:** 10.3390/ijerph191811395

**Published:** 2022-09-10

**Authors:** Jianwu Chen, Longzhe Jin, Bin Yang, Zhenfang Chen, Guoliang Zhang

**Affiliations:** 1 School of Civil and Resource Engineering, University of Science and Technology Beijing, Beijing 100083, China; 2 Institute of Occupational Health, China Academy of Safety Science and Technology, Beijing 100012, China

**Keywords:** lower exhaust hood, airflow distribution, uniform airflow, contamination control, workbench

## Abstract

Local exhaust ventilation is an important method of contamination control, and the type of exhaust hood and the air distribution at the hood face have an important influence on the contamination control effect. When the hood face is large, it is difficult to create a uniform airflow distribution at the hood face, which if achieved, could improve the effect of contamination control. To that end, the large-area workbench used in the process of vaccine purification was taken as the research subject prototype for this study. According to the methods for generating a uniform airflow distribution at the hood face, the lower exhaust workbenches of four structures were established using CAD and simulated using Ansys Fluent. The best uniformity of workbench surface air distribution was with Structure-4, while the worst was with Structure-1. The workbench surface airflow distribution could not achieve uniformity when only an inclined bottom was used for the large-area lower exhaust workbench with one side outlet. The more internal slits there were, the greater the air distribution area and the more uniform the air distribution. The width of the area of workbench surface airflow distribution was determined by the width of the slits. The numerical simulation results were verified by experiments, which showed them to be credible.

## 1. Introduction

Local exhaust ventilation is an effective method to control contaminants [1,2], and the exhaust hood type has an important influence on the contamination control effect [3]. When the lower exhaust hood is open, and its surface supports operation, workers can work around the lower exhaust workbench without influencing the setting of the exhaust hood. As the surface of the lower exhaust hood is close to contaminants, the collection efficiency is higher than that of other types of exhaust hoods, especially when the contaminant density is higher than that of air, and there is no heat source. Accordingly, the lower exhaust workbench has a wide range of applications, such as the purification of vaccines and polishing [4]. If the lower uniform air–exhaust hood is combined with a uniform air supply hood to create parallel push–pull ventilation, this has a better contamination control effect and a wider range of applications [5]. The distribution of velocity on the surface of the lower exhaust workbench is thus more uniform, while the contamination control effect [6,7,8] is better, and there is lower pressure loss [9,10,11] for the workbench. However, the larger the surface of the lower exhaust workbench, the more difficult it is to achieve a uniform airflow distribution at its surface, especially with an air outlet at the short side of the workbench.

The method of CFD simulation is widely used for this kind of application, and the 3D steady-state incompressible Navier–Stokes equations and the standard k-equation model are also used widely used when only momentum transfer is considered and heat transfer is ignored [12,13,14,15,16,17,18,19]. For example, the velocity change rules along the centerline outside the hood with a uniform airflow [12,13,14], the influence of the internal structure of the duct [15,16,17], the roof structure in a static pressure chamber [18] and the radius of the 90° rectangular elbow curvature in a lower exhaust hood [19] have each been studied in terms of airflow distribution using CFD simulations. Yet, the influence of the internal structure of a lower exhaust large-area workbench with an air outlet at the short side of the workbench has not been studied regarding surface airflow distribution. Therefore, a large-area workbench used in the process of vaccine purification was taken as the research subject in this study. This paper investigates the influence of the internal structure of a lower exhaust large-area workbench on its surface airflow distribution to better capture contaminants, which can be studied using the method of CFD simulation and verified using experiments.

## 2. Subjects and Methods

### 2.1. Geometric Models

#### 2.1.1. Models of the Workbench for Structural Research

**Figure 1:**
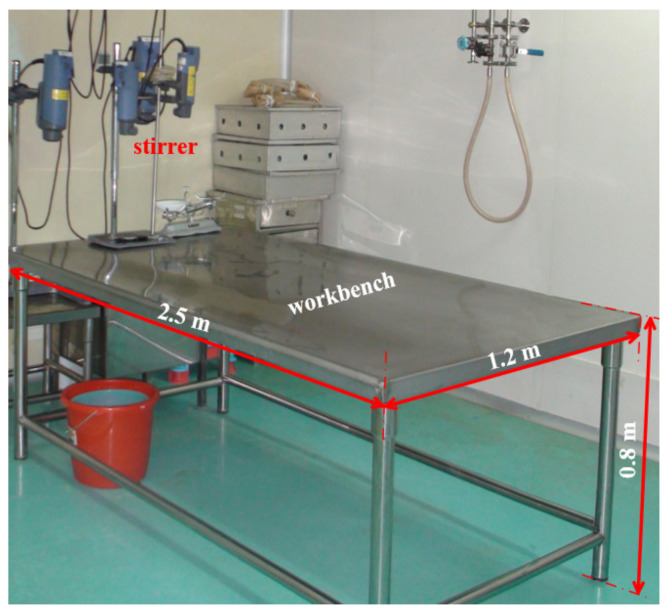
The large-area workbench prototype used in this study.

A large-area workbench used in the process of vaccine purification was taken as the research subject prototype for this study, with the following dimensions: length 2.5 m × width 1.2 m × height 0.8 m. The workbench is shown in Figure 1.

Workers add chloroform and vaccines to containers on both sides of the workbench and use stirrers to mix them, to inactivate and purify the vaccines. Large amounts of chloroform are used to purify the vaccines in this process, and this is volatilized into the air since the workbench lacks an exhaust, which is harmful to the occupational health of workers. To overcome this issue, a lower exhaust hood was added to the workbench to form a lower exhaust workbench, which can capture chloroform without affecting the operations of the workers.

**Figure 2:**
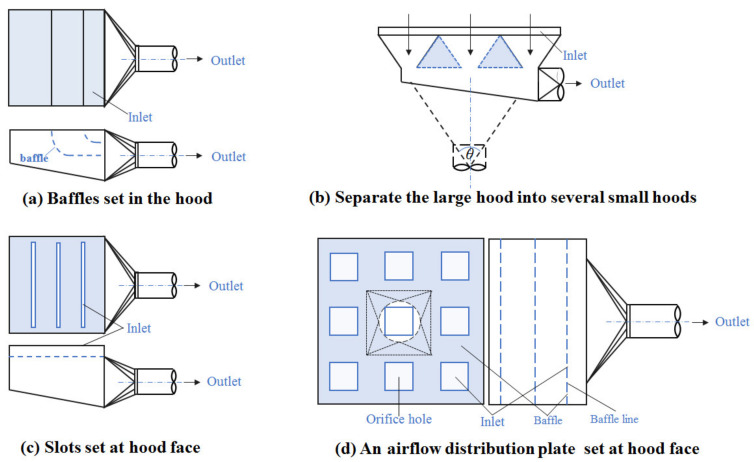
The methods for achieving a uniform airflow distribution at the hood face. *θ* is the inclination.

Due to limitations of the working space and operations, the contaminants captured by the exhaust hood can only be exhausted from one side outlet of the lower exhaust workbench, which means the velocity at its farthest point is 0 m/s, as shown in Refs. [18,19]. When the size of the hood face is large, it is difficult to achieve uniform airflow distribution on the hood face so the structures shown in Figure 2 can be used to improve airflow distribution [20]:

Figure 2a is to improve the airflow distribution at the hood face using baffles set in the hood. Figure 2b is to improve the airflow distribution at the hood face by setting triangular baffles in the hood to separate the large hood into several small hoods. Figure 2c is to set up slots at the hood face to realize the uniform airflow distribution at the slot; at this time, the velocity at the slot should be more than 10 m/s, and the velocity in the static pressure chamber should not exceed 1/2 of the slot velocity [20]. Figure 2d is to improve the airflow distribution by installing an airflow distribution plate at the hood face, such as the multi-hole orifice plate. 

**Figure 3:**
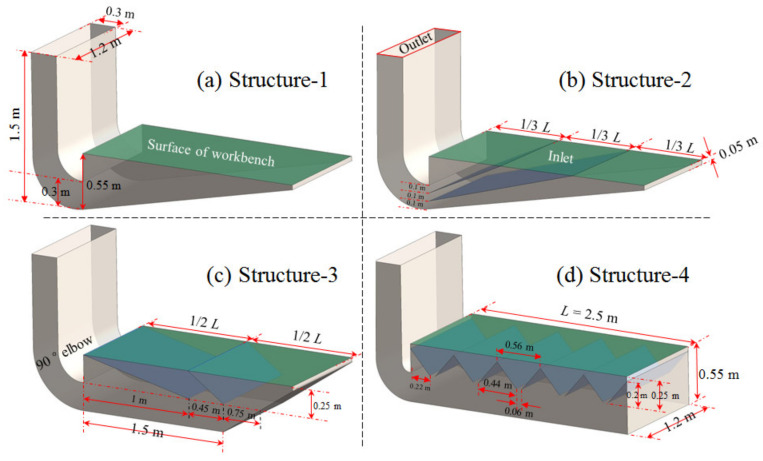
Four geometries of the lower exhaust workbench studied in this paper.

As can be seen from Figure 2a–c, an inclined bottom plate is used to achieve a uniform airflow distribution at the hood face, and it is also used to make the supply airflow uniform in the static pressure chamber of a spray room [18]. Therefore, the two geometries with bottom plate (as shown in Figure 3a,b) and two geometries with horizontal bottom plate (as shown in Figure 3c,d) were used for the study. In Figure 3c,d, the bottom plate structure type was studied for all or part of the bottom plate with a horizontal setting, which was used to study the geometric structure of different bottom plates.

According to Figure 2a, the hood face airflow distribution can be improved by setting baffles in the hood. Therefore, the geometries shown in Figure 3c,d were established to study the effect of the baffles in the hood and their direction on the airflow distribution at the hood face. According to Figure 2b, a model was established for dividing the hood into 5 small hoods by setting internal rectifier structures as shown in Figure 3d.

The principle shown in Figure 2c is only to realize the uniform airflow distribution on the slots, but there is no wind in the large area outside the slots, which is not consistent with the goal of uniform airflow distribution at the hood face. Therefore, the corresponding structural model was not established.

As shown in Figure 2d, an airflow distribution plate must be set at the surface of the lower exhaust workbench because it not only supports the operations, but it can also let contaminants pass through. If a uniform airflow distribution on the surface of the workbench can be achieved without an airflow distribution plate, this can then be further improved by adding an airflow distribution plate. Therefore, in order to study the effect of the inner structure of the workbench on the airflow distribution of its hood face, there was no airflow distribution plate set at the surface of workbench in the numerical simulation models, as shown in Figure 3.

According to the operation requirements, a height of 0.25 m was left under the workbench, an area that is used to set the discharge port and to remove residual liquid leaked from the surface of the workbench. The height of the lower exhaust workbench was set to 0.55 m without changing the height from the surface of the workbench to the ground. The length of the lower exhaust workbench was 2.5 m, and the width was 1.2 m, which was consistent with the workbench prototype. A length 1.2 m × width 0.3 m × height 1.5 m 90° elbow was set on the short side of the workbench, which was used to exhaust the contaminants captured by the lower exhaust workbench. The four structures of the lower exhaust workbench shown in Figure 3 were drawn using CAD.

#### 2.1.2. Model of the Workbench for Experimental Validation

**Figure 4:**
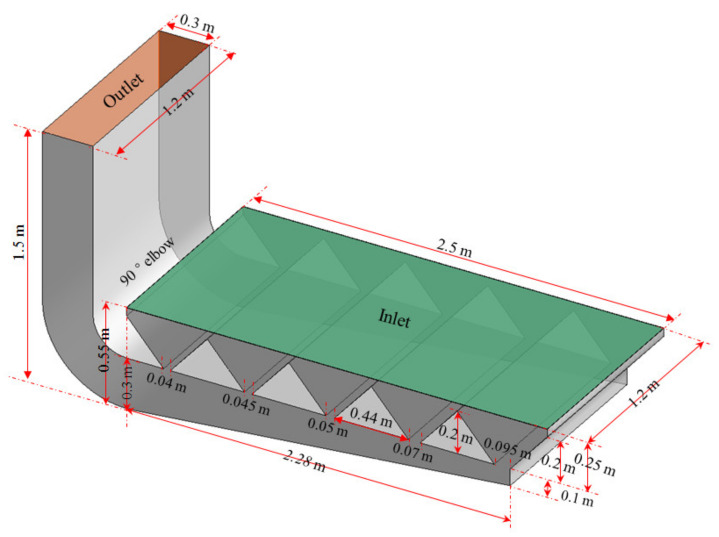
Lower exhaust workbench used for experimental validation.

According to the optimized results of workbench parameters, which will be discussed in other papers, an experimental lower exhaust workbench was established, as shown in Figure 4, which was used for experimental validation of the numerical simulation results.

The experimental workbench and the Structure-4 research workbench were the same except for the following differences:

(1) Although the method shown in Figure 2b was the same for both, the bottom plate was inclined in the experimental workbench, while it was horizontal in the numerical simulation workbench with Structure-4.
(2) Although both workbenches were set with five internal slits, the widths of the slits in the experimental workbench were 0.04 m, 0.045 m, 0.05 m, 0.07 m and 0.095 m from left to right, while they were all 0.06 m in the numerical simulation workbench with Structure-4.
(3) To eliminate the influence of the orifice plate on the airflow distribution, the multi-hole orifice plate was not set in the surface of the numerical simulation workbench with Structure-4, while it was set in the surface of the experimental workbench. The diameter of the holes in the multi-hole orifice plate was 2 mm, the spacing between the holes was 2 mm, and the arrangement of the holes was 60° staggered.

### 2.2. Research Conditions

To determine the optimal structure of the lower exhaust workbench with a uniform airflow distribution on its surface, four geometries of the workbench were simulated using Ansys Fluent.

The temperature of the airflow to the workbench was the same so heat transfer was ignored in this paper. Since the pressure at the workbench varied little, the gas was assumed to be incompressible. Only momentum transfer was considered. The standard *k*-epsilon model improved the accuracy of the calculation results by constraining the Reynolds stress, making the analysis of the airflow motion in the workbench more reliable. The SST *k*-epsilon model and the LES-type methods have been used to replace the standard *k*-epsilon model to solve the Navier–Stokes equation when only momentum transfer is considered and no heat transfer is taken into account. However, the standard *k*-epsilon model has been widely used [19] so the 3D steady-state incompressible Navier–Stokes equations and the standard *k*-epsilon dual equation model were used in this study. The settings for the solver in this study are shown in Table 1.

The aim of this study is to explore the influence of the internal structure of the workbench on its surface air distribution to find an internal structure with a good uniformity of surface air distribution. Therefore, the air distribution on the workbench surface with different internal structures could only be studied by setting the velocity at the air outlet of the elbow. So, the air outlet of the elbow was set as the inlet, and the surface of the lower exhaust workbench was set as the outlet.

When the surface velocity of the workbench is 0.6 m/s, it can meet the requirements for contamination control of the lower exhaust workbench when used as the uniform airflow exhaust hood in parallel with airflow push–pull ventilation [19]. According to the principle of air volume conservation, the velocity at the elbow outlet was 5 m/s in this study, which was calculated from Equation (1):

(1) $$v_{out}=v_{in}\times A_{in}/A_{out}\;$$

where:

$v_{in}$—Workbench surface velocity, m/s (0.6 m/s in this study).

$v_{out}$—Outlet velocity, m/s. Since the air was exhausted from the workbench, the outlet velocity was set to a negative value.

$A_{in}$—Workbench surface area (3 m^2^ in this study).

$\;A_{out}$—Area of the elbow outlet, m^2^ (0.36 m^2^ in this study).

The turbulence intensity (*I*) at the elbow outlet was 3.81%, as calculated from Equation (2):

(2) $$I=0.16Re^{\left(-1/8\right)}$$

where Re—Reynolds number. This was 9.59 × 10^4^, as calculated from Equation (3):

(3) Re=ρ·v·d/η

where:

ρ—Gas density, kg/m^3^. (The air was 1.205 kg/m^3^ at 20 °C in this study).

v—Hood face velocity, m/s. (At the outlet, it was 5 m/s in this study).

d—Hydraulic diameter, m (0.48 m for the outlet in this study).

η—Dynamic viscosity, Pa·s. (The air was 1.81 × 10^−5^ Pa·s at 20 °C in this study.)

Due to the airflow out of the workbench, it was necessary to set the velocity at the inlet, which is the velocity at the elbow outlet, to a negative value as shown in Table 2. Except for the workbench surface and the air exhaust outlet of the elbow, the rest of the surfaces in the model were set to the wall, and the settings of the boundary conditions for this study are shown in Table 2.

The Tet/Hybrid mesh method was used in ICEM, and the local grid was encrypted. Accurate grid division was ensured through the grid independence test. 

### 2.3. The Grid Sensitivity Tests

**Figure 5:**
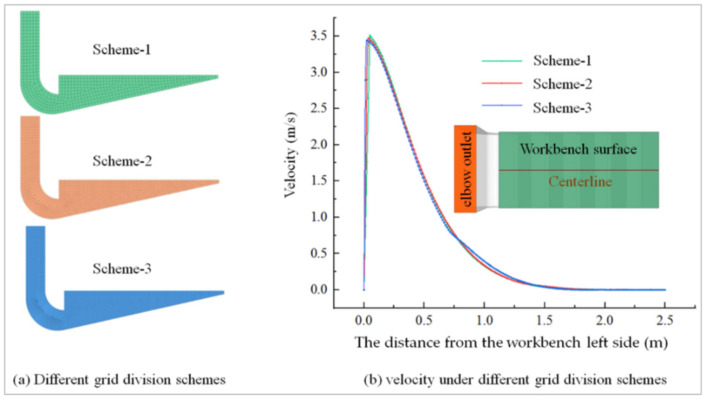
Variation of air velocity under different grid division schemes for Structure-1.

In the process of finite volume simulation, the quality of grids have an impact on simulation results, and it is necessary to ensure that the quality of grids meets the simulation requirements. Therefore, the workbench grid was checked for independence by taking the velocity variation of the workbench centerline as the index. Take Figure 3a Structure-1 as an example: the number of grid layers of the boundary layer was 5, and the growth rate was 1.2. Meshing was used for structured meshing, and the number of meshes for schemes 1, 2 and 3 was 13,776, 62,080 and 497,360, respectively, with dimensions of 50 mm, 30 mm and 15 mm. The simulated workbench centerline velocity obtained for Structure-1 is shown in Figure 5. It can be seen that the simulation results obtained from the three meshing schemes were similar, with little variability, and satisfied the finite element simulation requirements. However, due to the grid size, scheme 1 was larger; the features would be ignored in some locations with smaller sizes, while the grid number of scheme 3 was larger. Therefore, in consideration of the calculation time and simulation error, the grid drawn by scheme 2 was chosen for the subsequent simulation process.

The minimum orthogonal quality of Scheme 2 was 0.71, the maximum was 1, and the average was 0.98; the minimum skewness was 1.3 × 10^−10^, the maximum was 0.52, and the average was 0.055. Therefore, the grid quality of scheme 2 was better for Figure 3a, Structure-1.

### 2.4. Experimental Workbench and Test Methods 

**Figure 6:**
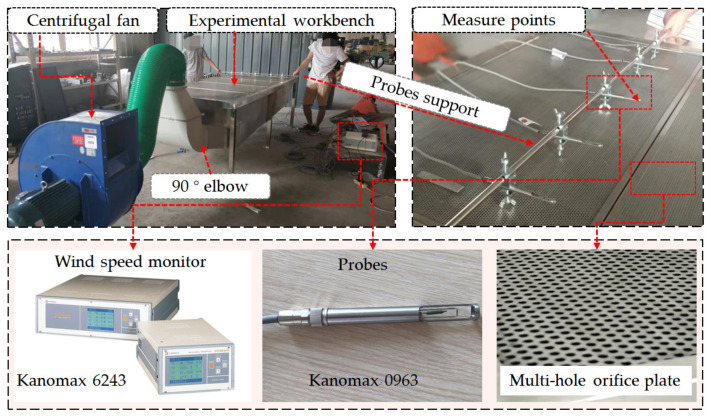
Experimental validation of lower exhaust workbench and measurement conditions.

According to the geometric model shown in Figure 4, a lower exhaust workbench was manufactured for the experimental research, as shown in Figure 6.

**Figure 7:**
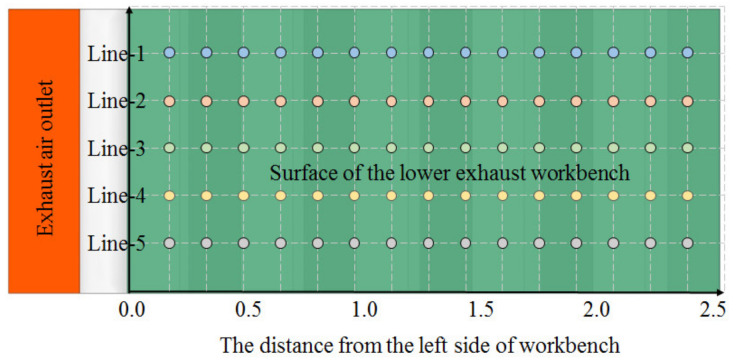
Layout of velocity measure points at the surface of the workbench.

The settings for the experimental lower exhaust workbench were the same as those for the numerical simulation workbench, as shown in Figure 7, except for the width and height of the elbow. The width of the elbow was as wide as the workbench in the numerical simulation, to reduce the influence of the elbow on airflow distribution. Meanwhile, the width of the elbow for the experimental workbench was 0.6 m, which was only half the width of the workbench. The centrifugal fan and the lower exhaust workbench were connected by the air duct.

Due to the height limit of the support probes, the velocities about 1 cm above the workbench surface were measured using an anemometer (Model 6243 with the probes of Model 0963, KANOMAX, Osaka, Japan); these were a little different from the velocity on the workbench surface. The velocities were measured at five points in the workbench short side direction and 15 points in the workbench long side direction, as shown in Figure 7. The velocities of the five points for the short side were measured at the same time, and the velocities for the 15 points for the length side were measured one by one. The measurement was performed for 100 results at 0.1 s intervals, and the average value was used as the result for each point.

## 3. Results

### 3.1. Velocity Contours for Differently Structured Workbenches

**Figure 8:**
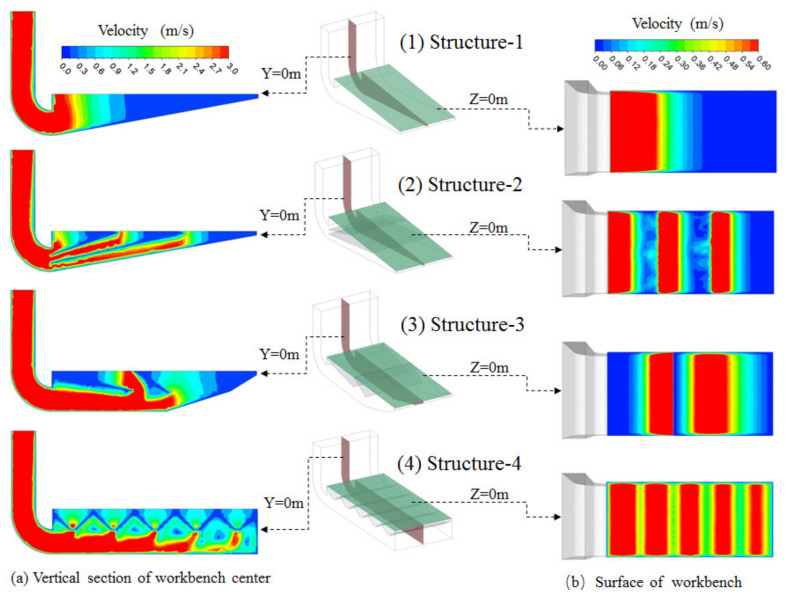
Velocity contour maps for differently structured workbenches. (**a**) Velocity contour map of a vertical section at the center of the workbench; (**b**) velocity contour map of the workbench surface.

Simulated velocity contour maps are shown in Figure 8.

As shown in Figure 8a, the uniformity of the airflow distribution on the workbench surface of Structure-1 was the worst in the four models studied in this paper. The velocity at the farthest point of the worktable was 0 m/s for the workbench of Structure-1, which is consistent with the results in reference [19]. The results show that it is difficult to achieve a uniform airflow distribution on the surface of the lower exhaust workbench by only setting the inclined bottom.

As can be seen from Figure 8, the airflow regional distribution numbers on the workbench surfaces of Structure-1, Structure-2, Structure-3 and Structure-4 were 1, 3, 2 and 5, respectively, which is consistent with the number of workbench surface divisions. So, the airflow regional distribution number at the surface of the lower exhaust workbench was determined by the number of workbench surface divisions. The more the workbench surface was divided, the greater the regional air distribution, meaning the air distribution on the workbench surface was more uniform. If the number of workbench surface divisions is large enough, the airflow on the surface of the workbench could be uniformly distributed. However, the best division number for the workbench surface area should meet the needs of a uniform air distribution and overcome the difficulties of processing, inspecting and maintaining the exhaust hood, which should be studied in the future.

The airflow distribution at the surfaces of the Structure-2 and Structure-3 workbenches did not meet the requirements for a uniform airflow distribution since the velocity at one end of the workbench surface was 0 m/s. Increasing the division number for the workbench surface by increasing the internal baffles of the workbench could improve the uniformity of the airflow at the surface of the workbench, but the height of the workbench limits the number of internal baffles. Therefore, these two geometries of the workbench are deemed unsuitable for the lower exhaust workbench studied in this paper.

Structure-4 was the most suitable structure for the lower exhaust workbench studied in this paper because the airflow distribution on the surface of the workbench with Structure-4 was the best of the four structures of workbenches studied. The velocity on the surface of the Structure-4 workbench presented five regional distributions. The velocity in the center of each region was the highest, and it was the lowest in the middle of the two slits. When the widths of the slits in the workbench were the same, the farther the slit was from the air exhaust outlet of the workbench, meaning the airflow distribution width of this region was smaller.

### 3.2. Workbench Surface Velocity for Differently Structured Workbenches

**Figure 9:**
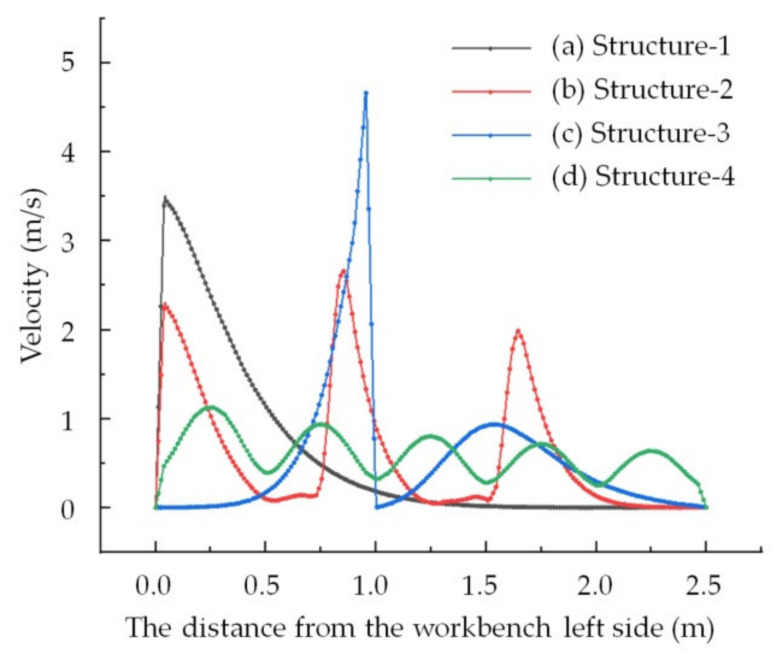
Workbench surface center velocity in the short side direction for differently structured workbenches studied in this paper.

As shown in Figure 8, the airflow distribution in the workbench short side direction was consistent, so the center velocity of the workbench short side direction could represent the velocity in this direction where the point is located. Accordingly, the center velocity in the workbench short side direction was used to analyze the airflow distribution in the workbench long side direction, and the simulation results for the velocities on the workbench surfaces for the four differently structured workbenches were plotted using the origin, as shown in Figure 9.

The workbench surface velocity of Structure-2 presented three regional distributions, and there was little difference in the maximum velocity between the three regions. The maximum velocity in each region rapidly decreased when increasing the distance from the center of the region, and the velocity between the two regions was about 0 m/s. The velocity on the left side of the workbench was too high, while it was too low on the other side.

The workbench surface velocity of Structure-3 presented two regional distributions. The uniformity of airflow distribution was worse than that of Structure-2, and the difference in the maximum velocity between the two regions was also bigger than that of Structure-2. The airflow distribution on the left side of the workbench surface was worse than that of Structure-2, but the airflow distribution on the right side was better. This shows that the internal baffle direction impacts the airflow distribution at the workbench surface. Meanwhile, the workbench surface airflow distribution of Structure-3 was the same as that of Structure-2, which was also unsuitable for the lower exhaust workbench studied in this paper.

The workbench surface velocity of Structure-4 presented five regional distributions with five internal slits evenly set, and the uniformity of the airflow was the best overall of the four differently structured workbenches. The maximum velocities in the five regions were largely the same and smaller than those in the other structures of workbenches. The maximum velocity decreased at the lowest rate with an increasing distance from the center of the region of the four structures of workbenches. Although the velocity difference between two regions was also smaller, the minimum velocities in the five regions were largely the same. The minimum velocity was still lower than the target value of 0.6 m/s, but it was bigger than that of the other structures of workbenches studied in this paper. So, the airflow uniformity of Structure-4 was the best of the four structures studied.

### 3.3. Velocity Vector Maps for Differently Structured Workbenches

**Figure 10:**
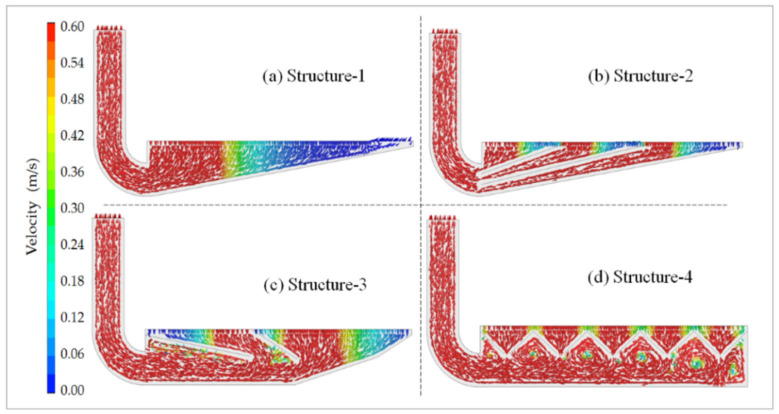
Velocity vector maps for four differently structured workbenches.

Based on the simulation results, the velocity vector maps for the four differently structured lower exhaust workbenches were created as shown in Figure 10.

As can be seen in Figure 10, the workbench surface airflow of Structure-1 was mainly distributed at the left side of the workbench, and the velocity was about 0 m/s at the right side of the workbench. The workbench surface airflow entered the workbench preferentially from the center of the region. The best uniformity of workbench surface air distribution was with Structure-4, the worst was with Structure-1, and Structure-2 and Structure-3 were in-between. Yet, eddy currents formed between the two slits in the lower exhaust workbench of Structure-4, which should be avoided. The matter should be studied in the future to determine how to avoid creating such eddy currents.

### 3.4. Experimental Validation Results

**Figure 11:**
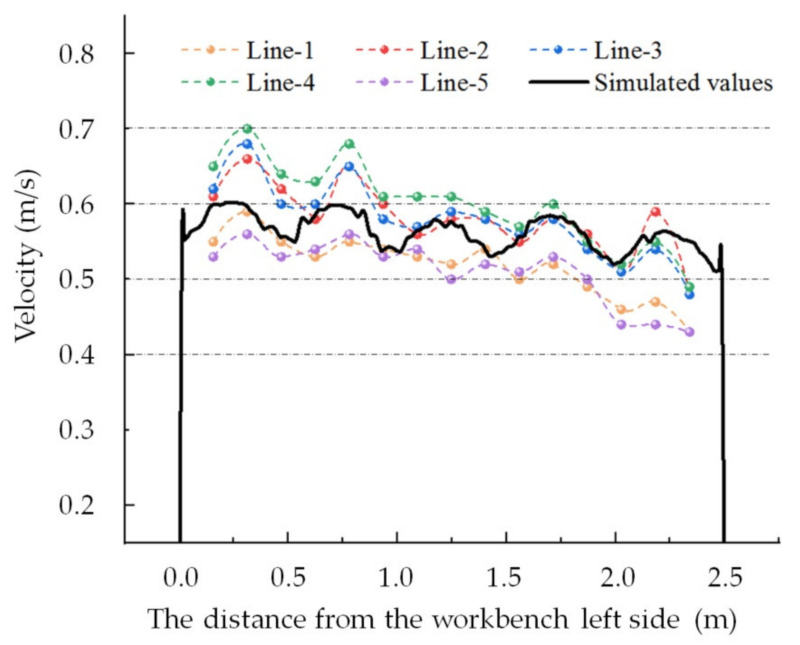
Measured velocities along five lines and simulated velocities along the centerline.

The same methods used for numerical simulation and setting boundary conditions in this paper were applied to simulate an experimental lower exhaust workbench as shown in Figure 4. As can be seen in Figure 8, the velocity in the workbench short side direction was consistent, while the velocity in the workbench long side direction greatly varied. Therefore, the numerical simulation results for the center velocity at the short side of the workbench can be taken to represent the velocity at the workbench short side, where the center velocity is located. The measured velocities along five lines were compared with the simulated workbench surface center velocities in the workbench short side direction, and the results are shown in Figure 11.

As shown in Figure 11, the simulated velocities were largely distributed between 0.5 m/s and 0.6 m/s for the lower exhaust workbench shown in Figure 4, reflecting a good uniformity of airflow distribution on the surface of the lower exhaust workbench with one side outlet. The measured velocities were almost equal to the simulated velocities, except for the velocities of Line-1 and Line-5 on the right side of the workbench, which were a little lower, and the velocities of Line-2, Line-3 and Line-4 on the left side of the workbench, which were a little higher. These differences may be due to the different widths of the elbow. The elbow for the experimental workbench was set in the middle of the workbench short side direction, while the width of the elbow was 50% of that in the numerical simulation of the workbench. This results in the high velocity near the center of the left of the workbench and the low velocity on both sides of the right of the workbench.

When measured and simulated, the average velocities were 0.558 m/s and 0.563 m/s, respectively, with a deviation of less than 1%. When measured and simulated, the maximum velocities were 0.638 m/s and 0.602 m/s, respectively, with a deviation of 5.9%. When measured and simulated, the minimum velocities were 0.463 m/s and 0.511 m/s, respectively, with a deviation of -9.58%. The numerical simulation results are thus largely consistent with the experimental results, which prove validity. 

The position of the velocity of the numerical simulation was on the workbench surface, while the position of the experimental measured velocity was about 1 cm above the workbench surface due to the limit on the support probes. The recorded deviations between the two results may have been related to the measurement height.

## 4. Discussion

The airflow distribution of the surface of the workbench could not be made uniform when only the inclined bottom was used in the large-area lower exhaust workbench, as shown in Figure 3a and proposed in this study. Yet, a uniform airflow distribution at the hood surface of the static pressure chamber could be achieved when only the inclined roof was set in a spray room, as proposed in reference [18]. The reason for the opposite results of the two research papers may be that one of the inclined bottom plates was used for the air exhaust and the other was used for the air supply. When the airflow was blocked by the inclined plate in the air supply ventilation, part of the dynamic pressure was converted to static pressure, to realize a uniform air supply. When the airflow was blocked by the inclined plate in the air exhaust ventilation, the airflow could get in from the other position with a lower static pressure since the airflow was mainly produced by the fan pressure in the air exhaust ventilation. The inclined plate showed little influence on the pressure at the surface of the exhaust hood, so the airflow distribution at the hood face was difficult to make uniform by only setting the inclined bottom plate. The bottom of the workbench was straight in Figure 3d, while it was inclined in Figure 4, and it is proposed that the influence of the inclined bottom plate on the workbench surface airflow distribution should be studied in the future.

The workbench surface airflow uniformities of Structure-2 and Structure-3 were worse than that of Structure-4, and they are not suitable for use in this lower exhaust workbench. This is mainly due to the height of the workbench, which limited the number and radian of internal baffles. If there was no limit on the height of the worktable, the workbenches with Structure-2 and Structure-3 could maybe also realize a uniform airflow distribution by setting a reasonable structure and suitable number of internal baffles.

The regional airflow distribution was related to the number of slits. The regional width of the airflow distribution on the surface of the worktable was determined by the width of the slits, as shown in Figure 8b,c. Figure 8b illustrates how if the width of the slits is the same, the further the distance is from the workbench air outlet and the smaller the airflow distribution width of the region. As shown in Figure 8c, the wider the width of the slits, the wider the airflow distribution width of the region. As shown in Figure 4, the closer the distance is to the workbench air outlet, the smaller the width of the slits, meaning a more uniform airflow distribution on the workbench surface could be achieved. In light of these findings, detailed studies should be carried out to confirm the structural parameters of the lower exhaust workbench, such as the number of slits, width of slits and diameter of the holes in the orifice plate.

## 5. Conclusions

The best uniformity of the workbench surface air distribution was with Structure-4, while the worst was with Structure-1, leaving Structure-2 and Structure-3 in-between. Yet, there were eddy currents between the two slits in the lower exhaust workbench of Structure-4, which should be avoided. Detailed studies should be carried out to confirm the structural parameters of the lower exhaust workbench, such as the number and width of slits.

The workbench surface airflow distribution could not be made uniform when only the inclined bottom was used in the large-area lower exhaust workbench with one side outlet. Yet, if changes are made to the exhaust to supply air, a uniform airflow distribution at the hood face could be achieved by only using the inclined roof.

The numbers of slits were 1, 2, 3 and 5 in the workbenches of Structure-1, Structure-3, Structure-2 and Structure-4, which were the same as the number of workbench surface divisions. Though the structures of the workbenches were different, the number of air distribution regions was related to the number of slits. The more slits there were, the more air distribution regions there were, meaning the air distribution was more uniform.

The regional width of the workbench surface airflow distribution was determined by the width of the slits. The wider the slit width, the wider the regional airflow distribution width. If the widths of internal slits were the same, the farther the slits were from the workbench air outlet, meaning the region airflow distribution width was smaller.

In this study, the numerical simulation results were largely consistent with the experimental results, which prove the numerical simulation results to be credible.

## Figures and Tables

**Table 1:** Solver parameter settings.

Solver Parameters	Parameter Settings
Solver type	Pressure-based
Solver velocity formulation	Absolute
Solver time	Steady
Viscous model	Standard *k*-epsilon
Species transport	Off
Energy	Off
Pressure–velocity coupling	SIMPLEC
Spatial discretization	Second order upwind
Convergence criterion	10^−6^
Interaction to plot and store	1000
Wall condition	Stationary wall/No slip

**Table 2:** Settings of boundary conditions.

Boundary Conditions	Parameter Settings
Outlet	Surface of lower exhaust workbench
Outlet boundary type	Pressure outlet
Gauge pressure (Pa)	0
Inlet	Air outlet of elbow
Inlet boundary type	Velocity inlet
Velocity at inlet (m/s)	–5
Material	Air
Air viscosity (kg/m/s)	1.81 × 10^−5^
Hydraulic diameter of inlet (m)	0.48
Turbulence intensity of inlet (%)	3.81

## Data Availability

The data presented in this study are available on request from the corresponding authors. The data are not publicly available due to privacy/ethical reasons.
